# Chemical characterization and biological activity in young sesame leaves (*Sesamum indicum* L.) and changes in iridoid and polyphenol content at different growth stages

**DOI:** 10.1371/journal.pone.0194449

**Published:** 2018-03-27

**Authors:** Yushiro Fuji, Ayumi Uchida, Katsunori Fukahori, Makoto Chino, Takashi Ohtsuki, Hiroshi Matsufuji

**Affiliations:** 1 Department of Food Bioscience and Biotechnology, College of Bioresource Sciences, Nihon University, Fujisawa, Kanagawa, Japan; 2 Wadaman Science Co., Ltd, Nijoden-cho, Nakagyo-ku, Kyoto, Japan; National Cancer Institute at Frederick, UNITED STATES

## Abstract

Three iridoids (lamalbid (**I1**), sesamoside (**I2**) and shanzhiside methyl ester (**I3**)) and seven polyphenols (cistanoside F (**P1**), chlorogenic acid (**P2**), pedalitin-6-*O*-laminaribioside (**P3**), pedaliin (**P4**), isoacteoside (**P6**), pedalitin (**P7**) and martynoside (**P8**)) were identified in young sesame leaves (*Sesamum indicum* L.) other than the acteoside (**P5**) reported previously. **P3** was a new compound, and **I1**, **I3**, **P2** and **P8** were found in a species of *Sesamum* for the first time. HPLC analyses revealed that the compounds **I1** (0.29–1.75% of dry leaves), **I2** (0.38–0.87%), **I3** (0.04–1.07%), **P4** (0.01–2.05%) and **P5** (0.13–4.86%) were present primarily in young sesame leaves and were found in plants cultivated on different farms (plant height, 30–70 cm). Of the identified compounds, **P5** and **P6** showed high 1,1-diphenyl-2-picrylhydrazyl (DPPH) radical scavenging, oxygen radical absorbance capacity (ORAC), and *in vitro* antiglycation activities. Given its content, **P5** makes a major contribution to the biological activities of young sesame leaves. The compounds were examined at six different growth stages of plants cultured in a greenhouse to determine the optimum harvest stage and for end-use assessment. **P5** accumulated in the leaves during growth, and the content reached a maximum of 12.9% of dry leaves in the 4th stage (plant height, 74.5±9.7 cm), which is one of the highest percentages reported in plants from nature.

## Introduction

Sesame (Pedaliaceae family, *Sesamum indicum* L.) is one of the most important oilseed crops and is widely cultivated in tropical and subtropical areas such as Myanmar, India, China, and Africa. In 2014, the production of sesame seeds was 6,235,530 t worldwide, with nearly 70% of the world production being in Asia and about 25% in Africa [1]. Sesame is harvested between 90 and 150 days after planting. About 65% of the annual crop is processed into oil and 35% is used in food [2]. The seeds contain lignans such as sesamin and sesaminol and are highly valued as a traditional health and nutraceutical food. The seed oil is used for cooking.

In contrast to sesame seeds, very little information is available on sesame leaves, probably because sesame is primarily cultivated for its seeds and the leaves are considered agricultural solid waste [3]. However, in Africa the leaves and shoots are used as vegetables, and the leaves contain useful amount of nutrients such as amino acids [4]. In China, decoctions of sesame leaves are used for various traditional and medicinal purposes, such as pain relief [5]. In Nigeria and many other tropical areas, the leaves of *Sesamum radiatum* are used for the treatment of catarrh, eye pain, bruises, and erupted skin lesions [6]. Given the potential physiological function of sesame leaves, the dried powder of young sesame leaves from plants 30–70 cm tall (about 40–60 days after planting) is commercially sold as a health food supplement in Japan.

Commercial products containing powdered young sesame leaves claim that the powder provides much more folic acid, potassium, riboflavin, and polyphenols than young barley leaves or kale. The Dictionary of Chinese Materia Medica, Japanese Edition [5] claims that the dried leaves of *S*. *indicum* contain 0.3% of the polyphenol pedaliin (6-hydroxy-luteolin 7-methyl ether 6-glucoside; pedalitin-6-*O*-glucoside), although no source is cited. Morita [7] reported the presence of pedaliin in sesame leaves based on its recrystallization from methanol extract, and Jain [8] reported 0.5% pedaliin in cultured sesame tissue. However, there are few reports regarding other polyphenol components in sesame leaves. Konan *et al*. [9] reported that the leaves of *S*. *radiatum* are rich in phenolic compounds, phenols, lignans, and flavonoids, and Kwak *et al*. [10] reported that the leaves of *S*. *indicum* show a higher total polyphenol content than do perilla leaves, although the composition and amount were not reported. Hata *et al*. [11] concluded that trace levels of lignans are present in *S*. *indicum* leaves (0.5–2.6 μg/g dry weight; 0.00005–0.00026%). We found that young sesame leaves contain more than eight polyphenols, with acteoside (**P5** in this study), a phenylethanoid, being one of the major components [12].

In this study, we identified three iridoids and seven polyphenols other than acteoside, and evaluated *in vitro* antioxidant (DPPH and ORAC) and antiglycation activity of the identified compounds. In addition, we investigated the content of major components in young sesame leaves from plants cultivated on different farms in Japan, and the change in composition of the leaves at six different growth stages to determine the optimum harvest stage and for end-use assessment. Finally, we identified and quantified the compounds in the leaves.

## Materials and methods

### Chemicals

Caffeic acid, hydroxytyrosol, chlorogenic acid (3-caffeoylquinic acid, **P2**), acteoside (verbascoside, **P5**), and isoacteoside (isoverbascoside, **P6**) were obtained from Sigma-Aldrich Co. (St. Louis, MO, USA), BP Biochemicals Inc. (San Diego, CA, USA) or ChromaDex Inc. (Irvine, CA, USA). DPPH (1,1-diphenyl-2-picrylhydrazyl) and aminoguanidine hydrochloride were from Wako Chemical Industries (Osaka, Japan), and fluorescein sodium salt, AAPH (2,2′-azobis(2-amidinopropane) dihydrochloride) and bovine serum albumin (BSA) were from Sigma-Aldrich Co. All reagents were of HPLC or special grade, purchased from Wako Chemical Industries, and were used without further purification. MeOH-*d*_4_ and DMSO-*d*_6_ were obtained from Isotech Inc. (Hatfield, PA, USA).

### Sesame leaf samples

The sesame variety Myanmar Black Sesame (Lignan-Rich Black Goma) was cultivated in private open-field farms in different regions (Kagoshima, Shimane, Kumamoto, and Miyazaki) in 2013–2016 in Japan, and young leaves were harvested at about 40–60 days after planting (plant height, about 30–70 cm) with the permission of the owners. This research field did not involve endangered or protected species. The leaves were washed with water, blanched for 60 s at 90 to 95 °C, dried for about 14 h at 40 to 60 °C, ground into a powder and passed through a 1.0 mm sieve, then dried with superheated steam to inactivate microorganisms. Dried powder of young sesame leaves was obtained from Wadaman Co. Ltd. (Kyoto, Japan).

Sesame plants were also grown in June 2015 (N.U. 2015) or from May to August 2016 (N.U. 2016) in a greenhouse at the College of Bioresource Sciences, Nihon University. For N.U. 2015, the leaves were collected from eight plants about 40 days after planting (plant height, about 30 cm). In N.U. 2016, the seeds were sown on May 9, 2016 in seed trays, and young seedlings were transplanted when plants reached the stage of 2–3 true leaves on May 22, 2016 (plant height, about 5 cm) to 3 L pots filled with red clay (1.5 L) and Metro-Mix-360 (1.5 L, Sun Gro Horticulture Canada, Vancouver, Canada) containing fertilizer (6 g/pot; N:P_2_O_5_:K_2_O:MgO = 6:40:6:15). The pots were put in an unheated greenhouse (variation in topsoil temperature throughout the experiment is presented in supporting information S1 Fig). Leaves were collected from 8–9 plants at six different growth stages (1st stage, June 7, 7.2±1.6 cm (*n* = 9); 2nd, June 30, 30.84.3 cm (*n* = 9); 3rd, July 14, 57.5±4.3 cm (*n* = 9); 4th, July 14, 74.5±9.7 cm (*n* = 8); 5th, July 21, 81.9±11.9 cm (*n* = 8); 6th, August 4, 125.9±4.3 cm (*n* = 8). Fresh and dry weight and number of leaves are summarized in supporting information S1 Table, and the studied growth stages are illustrated in S2 Fig). On July 14, the leaves were collected from two groups with or without buds and flowers. Any buds, flowers, capsules, or stem apices were removed to evaluate the chemical composition of leaves at various growth stages. The leaves were lyophilized and ground using a mortar and pestle. All samples of powder were kept at –20 °C until used.

### Extraction and isolation

Dried powder of young sesame leaves (0.5 kg) was defatted with *n*-hexane (1.0 L × 2), then extracted with 80% EtOH (1.0 L × 2) for 1 h at room temperature. This procedure was conducted three times to obtain 6.0 L of EtOH extracts from 1.5 kg of powder. The extracts were concentrated to 450 mL and the solution (150 mL × 3) was loaded onto an HP-20 column (5.5 × 53 cm) and eluted with MeOH (1.5 L × 3) to remove most of the chlorophyll. The MeOH effluent was evaporated *in vacuo*, and the residual aqueous solution was successively partitioned with EtOAc and *n*-BuOH. The *n*-BuOH phase was allowed to stand overnight at –20 °C, then the supernatant (Fr. BS, 55 g) and precipitate (Fr. BP, 5 g) were separated using filter paper. Half of Fr. BS was redissolved in 80% EtOH and heated at 80 °C for 24 h under mild alkaline conditions (pH 8.0 with 0.01 mol/L ammonium acetate) to hydrolyze most of the acteoside **(P5**) and obtain a mixture of **I1**, **I2** and **I3**. The other half of Fr. BS was loaded onto a Chemco LC-SORB ODS glass column (2.4 × 36 cm, Chemco Plus Scientific Co. Ltd., Osaka, Japan) and eluted with 70% MeOH containing 0.1% HCOOH to afford a brown fraction (Fr. BS-B). Isolation of each compound was performed using a preparative HPLC or TLC as follows, and the purity (%) of isolated compound was estimated by HPLC analysis.

The iridoids lamalbid (**I1**, 11 mg, 99%), sesamoside (**I2**, 8 mg, 98%) and shanzhiside methyl ester (**I3**, 11 mg, 99%) were isolated from the alkaline hydrolysate of Fr. BS using recycling preparative HPLC (Senshu Scientific SSI1300 instrument, Waters XBridge C18 column, 10 × 250 mm, 10–30% MeOH containing 0.1% HCOOH). The polyphenols cistanoside F (**P1**, 2.6 mg, 98%), pedalitin-6-*O*-laminaribioside (**P3**, 4.7 mg, 99%) and pedalitin (**P7**, 11 mg, 97%) were isolated from Fr. BS-B using recycling preparative HPLC (GL Science Inertsil PREP-ODS column, 20 × 250 mm or Waters XBridge C18 column, 10 × 250 mm, 30–40% MeOH containing 0.1% HCOOH). Pedaliin (pedalitin-6-*O*-glucoside, **P4**, 32 mg, 99%) was isolated from Fr. BP using recycling preparative HPLC (XBridge C18 column, 40% MeOH containing 0.1% HCOOH). Martynoside (**P8**, 7.6 mg, 99%) was isolated from Fr. BS using preparative TLC (Merck silica gel 60, CHCl_3_/MeOH/H_2_O (70:30:5 (by vol)), R_F_: 0.90) and recycling preparative HPLC (XBridge C18 column, 40% MeOH containing 0.1% HCOOH).

### HPLC or LC-ESI-MS/MS analysis

HPLC was conducted using an Agilent HP-1100 series instrument, a UV-Vis detector (Tokyo, Japan), and a Waters XBridge C18 column (4.6 × 150 mm, 5 μm) eluted with a linear MeCN gradient in 0.1% HCOOH (5 to 35% MeCN in 15 min, 35 to 100% MeCN in 25 min, and 100 to 5% MeCN in 1 min) at a flow rate of 0.8 mL/min at 40 °C. The sample injection volume was 10 μL and the eluent was monitored at 254 and 340 nm. For LC-ESI-MS/MS, a Waters Quattro Premier XL mass spectrometer coupled to an ACQUITY UPLC system with a Waters AQUITY photodiode array detector was used (Tokyo, Japan). Compounds were separated on a UPLC system with a Waters BEH C18 column (2.1 × 50 mm, 1.7 μm) and a linear MeCN gradient in 0.1% HCOOH (5 to 35% MeCN in 3 min, 35 to 100% MeCN in 5 min, and 100 to 5% MeCN in 1 min) at a flow rate of 0.3 mL/min at 40 °C. The sample injection volume was 1 μL. A triple quadrupole mass spectrometer with an electrospray ionization source was used for mass determination. The ion source conditions were optimized as follows: source temperature, 120 °C; capillary voltage, 3.5 kV; desolvation temperature, 400 °C; flow rate of desolvation gas, 850 L/h; flow rate of cone gas, 50 L/h. The mass spectrometer was operated in both the positive and negative ion modes, with a scan range from *m/z* 200 to 1200. For MS/MS experiments, different collision energies were used in the negative ion mode depending on the compounds studied.

### Identification of isolated compounds

High-resolution mass spectra were measured by direct infusion in electrospray negative ion mode using a Thermo Q-Exactive Orbitrap mass spectrometer (Waltham, MA, USA). ^1^H- and ^13^C-NMR spectra were measured using a JEOL ECA-500 spectrometer (Tokyo, Japan) at 500 MHz and 150 MHz, respectively in methanol-*d*_4_ or DMSO-*d*_6_ with tetramethylsilane as an internal standard. The signals in the ^1^H- and ^13^C-NMR spectra of the isolated compounds were assigned on the basis of chemical shifts and the results of 2D NMR (DQF-COSY, NOESY, HSQC-TOCSY, and HMBC) studies. Optical rotation was measured with a JASCO P-1020 polarimeter (Tokyo, Japan).

**I1**, lamalbid. White amorphous powder; UV-λmax: 237 nm; ${\left[\alpha \right]}_{D}^{26.0}$ –72.0° (I1.0, H_2_O); positive ESI-MS: *m/z* 867 [2M+Na]^+^, 445 [M+Na]^+^, 423 [M+H]^+^; negative ESI-MS: *m/z* 421 [M-H]^-^, 259 [aglycone]^-^; ^1^H- and ^13^C-NMR data, see S2 Table.

**I2**, sesamoside. White amorphous powder; UV- λmax: 232 nm; ${\left[\alpha \right]}_{D}^{26.1}$ –72.3° (c 0.58, MeOH); positive ESI-MS: *m/z* 863 [2M+Na]^+^, 443 [M+Na]^+^, 421 [M+H]^+^; negative ESI-MS: *m/z* 419 [M-H]^-^, 257 [aglycone]^-^; ^1^H- and ^13^C-NMR data, see S2 Table.

**I3**, shanzhiside methyl ester. White amorphous powder; UV-λmax: 237 nm; ${\left[\alpha \right]}_{D}^{25.3}$ –142.4° (c 0.38, MeOH); positive ESI-MS: *m/z* 835 [2M+Na]^+^, 430 [M+Na]^+^, 407 [M+H]^+^; negative ESI-MS: *m/z* 405 [M-H]^-^, 243 [aglycone]^-^; ^1^H- and ^13^C-NMR data, see S2 Table.

**P1,** cistanoside F. Yellowish amorphous powder; UV-λmax: 298, 331 nm; ${\left[\alpha \right]}_{D}^{25.3}$ –29.5° (I1.0, MeOH); positive ESI-MS: *m/z* 511 [M+Na]^+^, 489 [M+H]^+^, 471, 325, 163; negative ESI-MS: *m/z* 487 [M-H]^-^, 179 [M-Glc-Rhm-H]^-^; ^1^H- and ^13^C-NMR data, see S3 Table.

**P2**, chlorogenic acid (3-caffeoylquinic acid) (commercial authentic sample). UV-λmax: 297, 326 nm; positive ESI-MS: *m/z* 355 [M+H]^+^, 163; negative ESI-MS: *m/z* 353 [M-H]^-^, 191 [M-Caf]^-^.

**P4,** pedalitin-6-*O*-glucoside (pedaliin). Yellowish amorphous powder; UV-λmax: 271, 344 nm; ${\left[\alpha \right]}_{D}^{26.6}$ –4.3° (c 0.27, dioxane/EtOH = 1/9); positive ESI-MS: *m/z* 479 [M+H]^+^, 317 [M-Glc+H]^+^; negative ESI-MS: *m/z* 477 [M-H]^-^, 315 [M-Glc-H]^-^; ^1^H- and ^13^C-NMR data, see Table 1.

**Table 1:** NMR spectroscopic data for compounds P3, P4 and P7.

pos.	P3 (DMSO-*d*_6_)		P4 (DMSO-*d*_6_)		P7 (DMSO-*d*_6_)	
	δ _C_	δ _H_ (mult, *J* in Hz)	δ _C_	δ _H_ (mult, *J* in Hz)	δ _C_	δ _H_ (mult, *J* in Hz)
2	164.0		164.0		163.8	
3	102.6	6.75 (s)	102.6	6.74 (s)	102.3	6.70 (s)
4	182.0		182.0		181.9	
4a	104.7		104.7		104.8	
5	151.5		151.5		146.0	
6	127.8		127.9		129.7	
7	158.2		158.3		154.1	
8	91.4	6.89 (s)	91.4	6.88 (s)	90.9	6.87 (s)
8a	152.4		152.4		149.4	
1'	121.3		121.3		121.5	
2'	113.4	7.45 (s)	113.4	7.45 (s)	113.3	7.45 (s)
3'	145.6		145.6		145.6	
4'	149.6		149.6		149.5	
5'	115.8	6.90 (d, 9.0)	115.8	6.90 (d, 8.0)	115.8	6.90 (d, 8.0)
6'	118.9	7.46 (d, 9.0)	118.9	7.46 (d, 8.0)	118.7	7.44 (d, 8.0)
7-OCH_3_	56.4	3.92 (s)	56.4	3.91 (s)	56.1	3.92 (s)
5-OH		13.1 (s)		13.1 (s)		12.7 (s)
GlcA-1''	101.0	5.16 (d, 7.0)	101.8	5.05 (d, 7.0)		
2''	72.8	3.47 (m)	74.0	3.22 (m)		
3''	87.6	3.45 (m)	76.4	3.22 (m)		
4''	68.0	3.30 (m)	69.8	3.12 (m)		
5''	76.7	3.20 (m)	77.1	3.06 (m)		
6''	60.5	3.60 (brd, 10.5)	60.7	3.60 (dd, 3.0, 11.0)		
		3.45 (m)		3.41 (m)		
GlcB-1‴	103.9	4.35 (d, 8.0)				
2‴	73.7	3.08 (m)				
3‴	75.9	3.21 (m)				
4‴	70.0	3.05 (m)				
5‴	76.8	3.18 (m)				
6‴	61.0	3.70 (brd, 11.0)				
		3.37 (m)				

**P5,** acteoside (commercial authentic sample). UV-λmax: 293, 335 nm; ${\left[\alpha \right]}_{D}^{25.9}$ –85.2° (I1.0, MeOH); positive ESI-MS: *m/z* 647 [M+Na]^+^, 625 [M+H]^+^, 471, 163; negative ESI-MS: *m/z* 623 [M-H]^-^.

**P6,** isoacteoside (commercial authentic sample). UV-λmax: 292, 327 nm; ${\left[\alpha \right]}_{D}^{26.7}$ –40.5° (c 0.45, MeOH); positive ESI-MS: *m/z* 647 [M+H]^+^, 625 [M+H]^+^, 325, 163; negative ESI-MS: *m/z* 623 [M-H]^-^, 461 [M-Caf]^-^, 161.

**P7**, pedalitin. Yellowish amorphous powder; UV-λmax: 282, 344 nm; positive ESI-MS: *m/z* 317 [M+H]^+^, 302; negative ESI-MS: *m/z* 315 [M-H]^-^, 300; ^1^H- and ^13^C-NMR data, see Table 1.

**P8**, martynoside. Yellowish amorphous powder; UV-λmax: 288, 330 nm; ${\left[\alpha \right]}_{D}^{26.0}$ –45.3° (I1.0, MeOH); positive ESI-MS: *m/z* 653 [M+H]^+^, 485, 339; negative ESI-MS: *m/z* 651 [M-H]^-^; 475, 193, 175; ^1^H- and ^13^C-NMR data, see S3 Table.

### Content of iridoids and polyphenols

The concentration of each compound in sesame leaves was measured by HPLC. Dry sesame leaf powder was extracted twice with 1 mL (for ~25 mg samples) or 50 mL 60% MeOH (for ~100 mg samples) using a sonicating bath (15 min). After passing the extracts through a 0.45 μm PVDF membrane filter, the filtrate was subjected to HPLC. Calibration curves were constructed using authentic or isolated compounds (0.005–0.5 mg/mL). The amount of each analyzed compound in the leaves was expressed as percentage of dry leaf weight.

### Antioxidant activity

DPPH assays were performed according to our previous report [12] with modifications to allow the use of 96-well plates. Authentic and isolated compounds were dissolved in 60% MeOH (except **P4**, which was dissolved in DMSO) and diluted with 60% MeOH as necessary. Standard (Trolox, 20 μL) or sample (20 μL) was mixed with 80 μL 100 mmol/L Tris-HCl buffer (pH 7.4) and 100 μL 0.20 mmol/L DPPH in EtOH solution in a 96-well plate. The decrease in absorbance was monitored at 520 nm for 30 min using a Tecan microplate spectrophotometer (Kanagawa, Japan). Results are expressed as μmol Trolox equivalent (TE)/g or mol TE/mol.

Oxygen radical absorbance capacity (ORAC) assays were performed according to the method of Watanabe *et al*. [13] with some modifications. Authentic and isolated compounds were dissolved in acetone/water/acetic acid (70:29.5:0.5 (by vol); AWA) and diluted with assay buffer (75 mmol/L KH_2_PO_4_/K_2_HPO_4_, pH 7.4) as necessary. Standard (Trolox, 20 μL) or sample (20 μL) was mixed with 200 μL 94.4 nmol/L sodium fluorescein solution, preincubated at 37 °C for 5 min in a 96-well plate, then incubated with 31.7 μmol/L AAPH solution (75 μL) at 37 °C. The fluorescence (excitation, 485 nm; emission, 528 nm) was monitored every 2 min for 90 min using a Bio-Tek Synergy 2 multi-mode microplate reader (Winooski, VT, USA). The net area under the curve (AUC) was calculated by subtracting the AUC for the blank from that of each sample. The ORAC value for each sample was calculated using a standard curve for Trolox (6.25, 12.5, 25, and 50 μmol/L). Results are expressed as μmol TE/g or mol TE/mol.

### Antiglycation activity

Inhibition of BSA glycation *in vitro* was assayed according to the method of Tsuji-Naito *et al*. [14] with some modifications. BSA (10 mg/mL) was mixed with 50 mmol/L glucose and fructose in 50 mmol/L phosphate buffer solution (pH 7.4) under sterile conditions. The solution (1.5 mL) was incubated in the presence or absence (as a control) of isolated compounds in water or DMSO (0.001 to 0.015 mg/mL in the final) for 6 days at 37 °C in the dark. For the blank, phosphate buffer was used instead of sugar solution. The degree of BSA glycation was measured using a JASCO FP-6500 spectrofluorometer (Tokyo, Japan) at an excitation wavelength of 370 nm and an emission wavelength of 440 nm. The inhibitory activity (%) was calculated as follows:

$$Inhibitory\;activity\;\left(\%\right)=\left[\left(FL_{control}-FL_{blank}\right)–\left(FL_{sample}-FL_{blank}\right)/\left(FL_{control}-FL_{blank}\right)\right]\times 100$$

The concentration (μmol/L) of samples required to inhibit 50% of BSA glycation is defined as the IC_50_ value.

## Results and discussion

### Identification of iridoids and polyphenols in young sesame leaves

**Fig 1:**
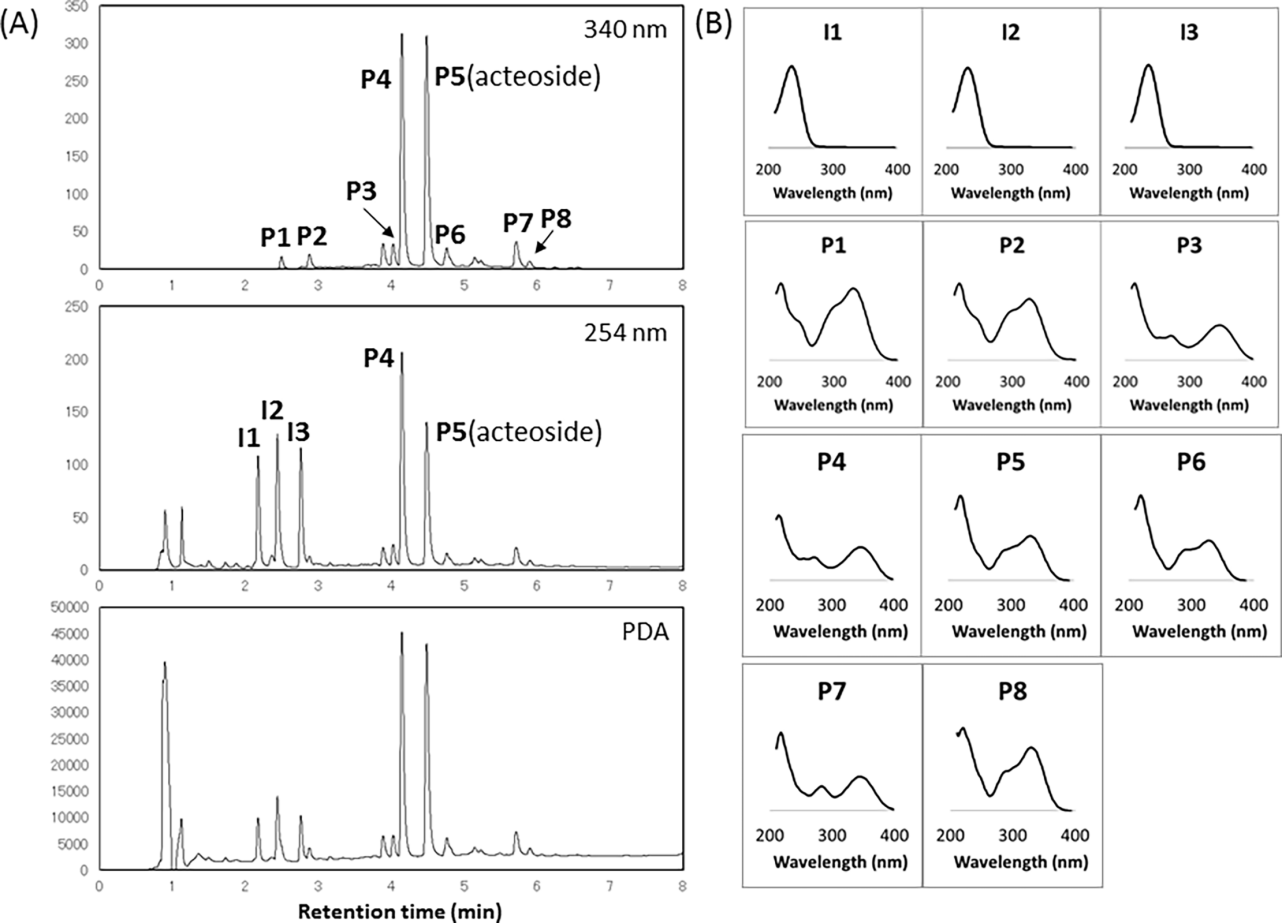
UPLC-PDA chromatograms (A) and UV spectral data (B) of young sesame leaves.

Typical HPLC chromatograms and photodiode array spectra of young sesame leaves are shown in Fig 1. Similar spectra were obtained for compounds **I1**, **I2**, and **I3**, for **P1**, **P2**, **P5**, **P6**, and **P8**, and for **P3**, **P4**, and **P7**, suggesting that the compounds in each of these groups have similar chromophores. Based on the chemical structure of acteoside (**P5**), we first attempted to identify the unknown compounds using available authentic caffeoyl derivatives and hydroxytyrosol. The structures of **P2** and **P6** were identified as chlorogenic acid (3-caffeoylquinic acid) and isoacteoside, respectively, based on comparison of retention times, UV and LC-ESI-MS spectra with authentic compounds. On the other hand, no caffeic acid or hydroxytyrosol was detected in young sesame leaves, even in analysis by multiple reaction monitoring methods with LC-ESI-MS/MS.

**Fig 2:**
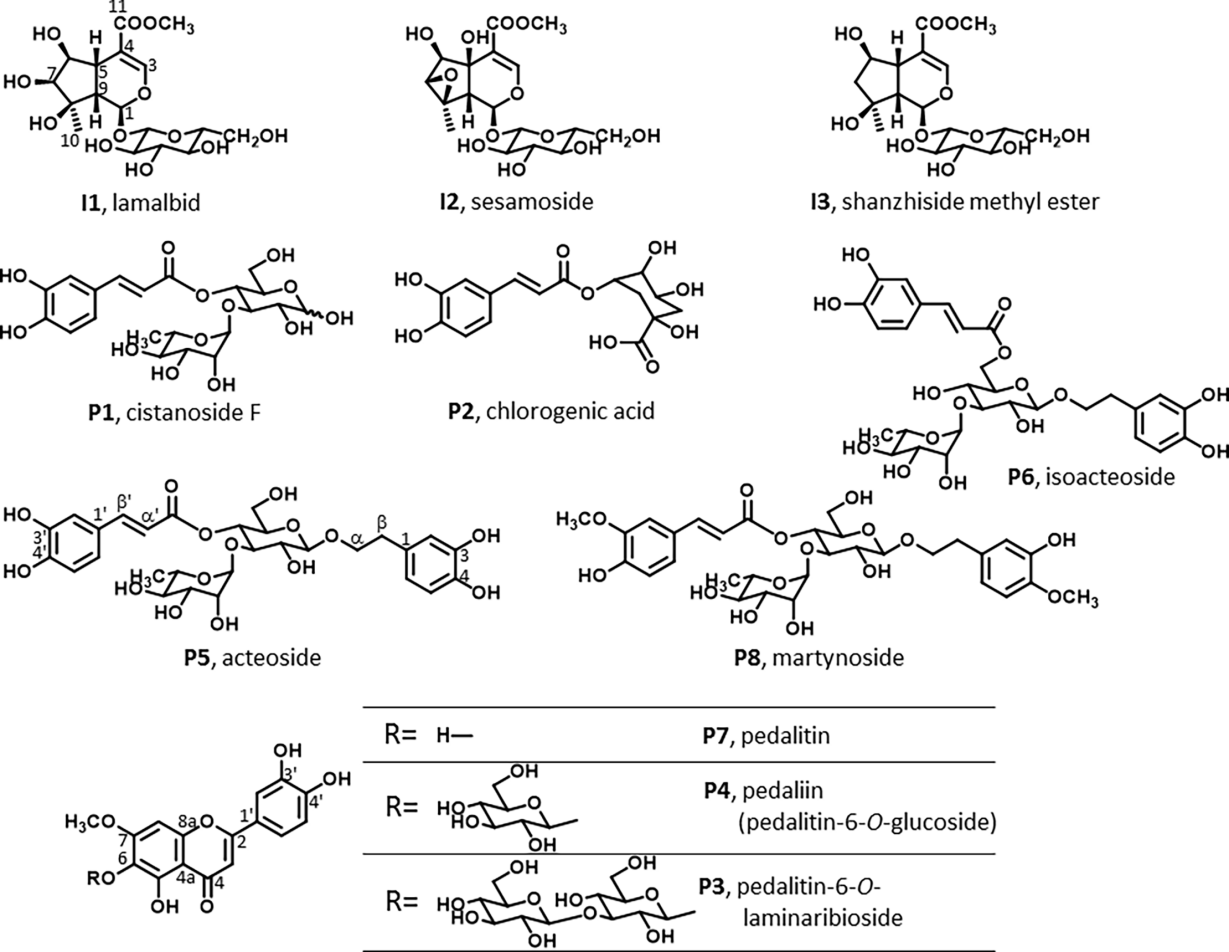
Iridoids and polyphenols found in young sesame leaves.

For the compounds isolated in this study, the LC-ESI-MS and the ^1^H- and ^13^C-NMR data of **I1**, **I2**, **I3**, **P1**, **P4**, **P7** and **P8** were shown to be identical to those of lamalbid, sesamoside, shanzhiside methyl ester [15–17], cistanoside F [18], pedaliin (pedalitin-6-*O*-glucoside), pedalitin [7,19] and martynoside [20,21], respectively (Fig 2).

Compound **P3** was isolated as a yellowish amorphous powder; UV-λmax: 271, 344 nm; ${\left[\alpha \right]}_{D}^{26.6}$ –19.6° (I1.0, dioxane/EtOH = 1/9). The LC-ESI-MS data obtained in positive and negative modes for compound **P3** showed molecular-related ion peaks at *m/z* 641 [M+H]^+^ and 639 [M-H]^-^, respectively. Also, high-resolution-ESI-MS (negative) ([M-H]^-^) data of *m/z* 639.1583 (calcd for I2_8_H_31_O_17_; 639.1560) indicated that **P3** has a molecular formula of I2_8_H_32_O_17_. Compared to pedalitin (**P7**) and pedaliin (**P4**), the molecular weight of **P3** was 324 and 162 mu larger, respectively. In addition, a peak corresponding to **P7** appeared when isolated **P4** or a mixture containing **P3** was hydrolyzed under acidic conditions. These findings suggested that **P3** is the diglycoside of pedalitin.

The ^1^H- and ^13^C-NMR data of **P3** suggested the presence of pedalitin and two sugar units (Table 1; NMR spectral data are shown in supporting information S3 Fig). The typical coupling constants (7.0 and 8.0 Hz) of the anomeric proton signals at δ 5.16 and δ 4.35, respectively, and the results of 2D-NMR (HSQC-TOCSY and NOESY) experiments, suggested that the two sugar units were β-form of glucopyranosides. Also, glycosidation-induced ^13^C NMR shifts in the A ring of the aglycone when compared with **P7** were observed, and similar chemical shifts were also observed for **P4**. Furthermore, correlations between H-1'' of glucose A (the anomeric proton signal at δ 5.16) and C-6 of the pedalitin unit (δ 127.8) were observed in the HMBC spectrum. In addition, correlation between H-1‴ of glucose B (δ 4.35) and I3'' of glucose A (δ 87.6) was observed, suggesting that laminaribiose unit would be glycosylated to C-6 of the pedalitin unit. There are several reports of flavonoid glycosides containing a laminaribiose moiety, and the NMR data for the laminaribiose moiety of **P3** were almost the same as the data in these previous reports [22,23]. We therefore concluded that **P3** is pedalitin-6-*O*-laminaribioside (Fig 2). To the best of our knowledge, pedalitin-6-*O*-laminaribioside is a new compound.

Potterat *et al*. [16] reported the isolation of acteoside (**P5**), sesamoside (**I2**) and other iridoids (phlomiol, pulchelloside-I, and 6β-hydroxyipolamiide) from the root bark of *Sesamum* species (*S*. *angolense* W.), and Takase *et al*. [24] identified an iridoid, sesinoside, in illuminated seedlings of *S*. *indicum* L. Suzuki *et al*. [25] reported obtaining cistanoside F (**P1**), acteoside (**P5**), isoacteoside (**P6**), decaffeoyl acteoside, and campneoside I and II from a water extract of *S*. *indicum* whole plants, although they did not mention whether the harvested plants contained leaves, stems, roots, flowers, or seeds, nor the age at harvest. The various iridoids and polyphenols are presumably produced in different parts of sesame plants at different growth stages. Of the compounds identified in this study, **I1**, **I3**, **P2** and **P8** were found in a species of *Sesamum* for the first time.

### Iridoid and polyphenol contents of young sesame leaves cultivated on several farms

In general, it is well-known that the content of secondary compounds produced in plants varies with the development stage of the plant and between leaves, stems and roots, and also depends on environmental factors such as time of day, weather, and soil nutrient conditions. Thus, chemical components were examined in young leaves cultivated on different farms and in a greenhouse. Typical HPLC chromatograms obtained are shown in supporting information S4 Fig. No other large peaks corresponding to unidentified compounds were observed in any chromatograms. The composition of the leaves is summarized in Table 2. Lamalbid (**I1**), sesamoside (**I2**), shanzhiside methyl ester (**I3**), pedaliin (**P4**), and acteoside (**P5**) were major compounds in all young sesame leaves used in this study, although the content differed depending on the region and harvest year: **I1**, 0.29–1.75%; **I2**, 0.38–0.87%; **I3**, 0.04–1.07%; **P4**, 0.01–2.05%; **P5**, 0.13–4.86%. Olivier *et al*. [26] reported that climate and geology did not play a major role in the production of acteoside or isoacteoside in *Lippia javanica* from different localities. The factors determining production of secondary compounds in sesame leaves and the losses in sterilization processes are still being evaluated, and further investigations are needed before harvesting young sesame leaves for use as a health food supplement.

**Table 2:** Content (%) of compounds in young sesame leaves cultivated in different regions in harvest years 2013 through 2016.

	2013			2014	2015
	Kagoshima	Kumamoto	Shimane	Kagoshima	Kagoshima
**I1**	0.35 ± 0.009	0.89 ± 0.199	0.69 ± 0.006	0.62 ± 0.199	0.45 ± 0.199
**I2**	0.57 ± 0.009	0.74 ± 0.115	0.85 ± 0.017	0.96 ± 0.115	0.38 ± 0.115
**I3**	0.17 ± 0.005	0.12 ± 0.028	0.69 ± 0.012	0.62 ± 0.028	0.23 ± 0.028
**P1**	0.03 ± 0.002	0.02 ± 0.006	0.04 ± 0.001	0.01 ± 0.006	0.01 ± 0.006
**P2**	0.06 ± 0.001	0.12 ± 0.010	0.03 ± 0.001	0.01 ± 0.010	0.00 ± 0.010
**P3**	0.16 ± 0.004	0.10 ± 0.009	0.05 ± 0.003	0.03 ± 0.009	0.03 ± 0.009
**P4**	1.66 ± 0.038	1.00 ± 0.062	0.41 ± 0.011	0.16 ± 0.062	0.33 ± 0.062
**P5**	1.20 ± 0.039	2.47 ± 0.133	1.16 ± 0.027	0.54 ± 0.017	0.13 ± 0.017
**P6**	0.27 ± 0.012	0.22 ± 0.017	0.09 ± 0.002	0.03 ± 0.001	0.04 ± 0.001
**P7**	n.d.^c^	0.01 ± 0.001	0.01 ± 0.001	n.d.	0.02 ± 0.002
**P8**	n.d.	0.09 ± 0.013	0.04 ± 0.001	0.01 ± 0.000	0.01 ± 0.001
	2015	2016			
	N.U.^a^	Kagoshima	Shimane	Miyazaki (1)^b^	Miyazaki (2)^b^
**I1**	0.15 ± 0.019	0.55 ± 0.014	0.29 ± 0.002	1.37 ± 0.002	1.75 ± 0.009
**I2**	0.58 ± 0.046	0.51 ± 0.073	0.58 ± 0.004	0.50 ± 0.004	0.87 ± 0.001
**I3**	0.54 ± 0.008	0.11 ± 0.015	0.04 ± 0.001	0.67 ± 0.001	1.07 ± 0.004
**P1**	0.02 ± 0.000	0.03 ± 0.001	0.07 ± 0.001	0.02 ± 0.001	0.03 ± 0.000
**P2**	0.26 ± 0.001	0.03 ± 0.000	0.02 ± 0.000	n.d.	0.00 ± 0.000
**P3**	0.08 ± 0.004	0.07 ± 0.000	0.13 ± 0.001	0.04 ± 0.001	0.05 ± 0.000
**P4**	2.05 ± 0.094	0.01 ± 0.001	0.70 ± 0.002	0.37 ± 0.002	0.60 ± 0.002
**P5**	4.86 ± 0.047	1.32 ± 0.005	0.46 ± 0.004	0.13 ± 0.004	0.19 ± 0.002
**P6**	0.31 ± 0.030	0.02 ± 0.003	0.05 ± 0.002	0.01 ± 0.002	0.03 ± 0.001
**P7**	n.d.	0.32 ± 0.002	0.02 ± 0.001	n.d.	n.d.
**P8**	n.d.	0.02 ± 0.001	0.04 ± 0.001	n.d.	n.d.

^a^ N.U. was cultured in a greenhouse at Nihon University, Kanagawa and others were done on open-field farms in several regions.

^b^ Miyazaki (1) and (2) were cultured at different area in Miyazaki prefecture.

^c^ n.d., not detected.

### Antioxidant and antiglycation activities of iridoids and polyphenols in young sesame leaves

Table 3 summarizes the antioxidant and antiglycation activities of each of the iridoids and polyphenols isolated from young sesame leaves. Of the compounds, acteoside (**P5**) and isoacteoside (**P6**) had high DPPH radical scavenging activity, ORAC activity, and inhibitory activity of BSA glycation, while iridoids (**I1**, **I2** and **I3**) had no antioxidant and antiglycation activities. There are plenty of reports on the antioxidant and antiglycation activities of the compounds identified in this study, particularly acteoside and isoacteoside [27–32], and the activities we observed were almost similar to previous reports. Given the amount present of the compounds and their activities, acteoside (**P5**) makes a major contribution to the antioxidant and antiglycation activities of young sesame leaves. In addition, several researchers have reported that acteoside inhibit the formation of advanced glycation end products (AGEs) *in vivo* [31, 33]. Thus, young sesame leaves with a high content of acteoside likely inhibit the formation of AGEs, although further investigation is needed to verify the antiglycation effect *in vivo*.

**Table 3:** Antioxidant and antiglycation activities of the isolated compounds from sesame leaves.

	DPPH	ORAC	Inhibition of BSA glycation
	TEAC (μmol TE/g)	TEAC (μmol TE/g)	IC_50_ value (μM)
**I1**	n.a.^a^	n.a.	n.a.
**I2**	n.a.	n.a.	n.a.
**I3**	n.a.	n.a.	n.a.
**P1**	4100 ± 310 [2.85]^b^	11360 ± 220 [5.55]	345.1 ± 42.2
**P2**	3780 ± 290 [1.34]	22600 ± 2280 [8.01]	28.1 ± 0.06
**P3**	2020 ± 77 [1.29]	8210 ± 1200 [5.26]	132.7 ± 15.0
**P4**	2010 ± 130 [0.96]	24410 ± 1760 [9.29]	41.8 ± 0.57
**P5**	5470 ± 120 [3.41]	23600 ± 3480 [14.73]	10.0 ± 1.2
**P6**	5700 ± 590 [3.56]	18520 ± 2190 [11.57]	11.1 ± 0.14
**P7**	2030 ± 57 [0.64]	12380 ± 310 [3.92]	159.7 ± 2.4
**P8**	910 ± 59 [0.60]	8240 ± 260 [4.73]	n.a.
CA^c^	16230 ± 620 [2.93]	36560 ± 1910 [6.59]	117.6 ± 9.3
HT^c^	12180 ± 300 [2.20]	45310 ± 1480 [8.10]	44.2 ± 4.0
AG^c^	–	–	549.7 ± 74.8

The assays were carried out in at least triplicate and results are presented as means ± SD (*n* = 3 or 6).

^a^ n.a., no activity detected.

^b^ The value in box brackets shows TEAC (mol TE/mol).

^c^ CA, caffeic acid; HT, hydroxytyrosol; AG, aminoguanidine.

### Changes in chemical composition at different growth stages

**Fig 3:**
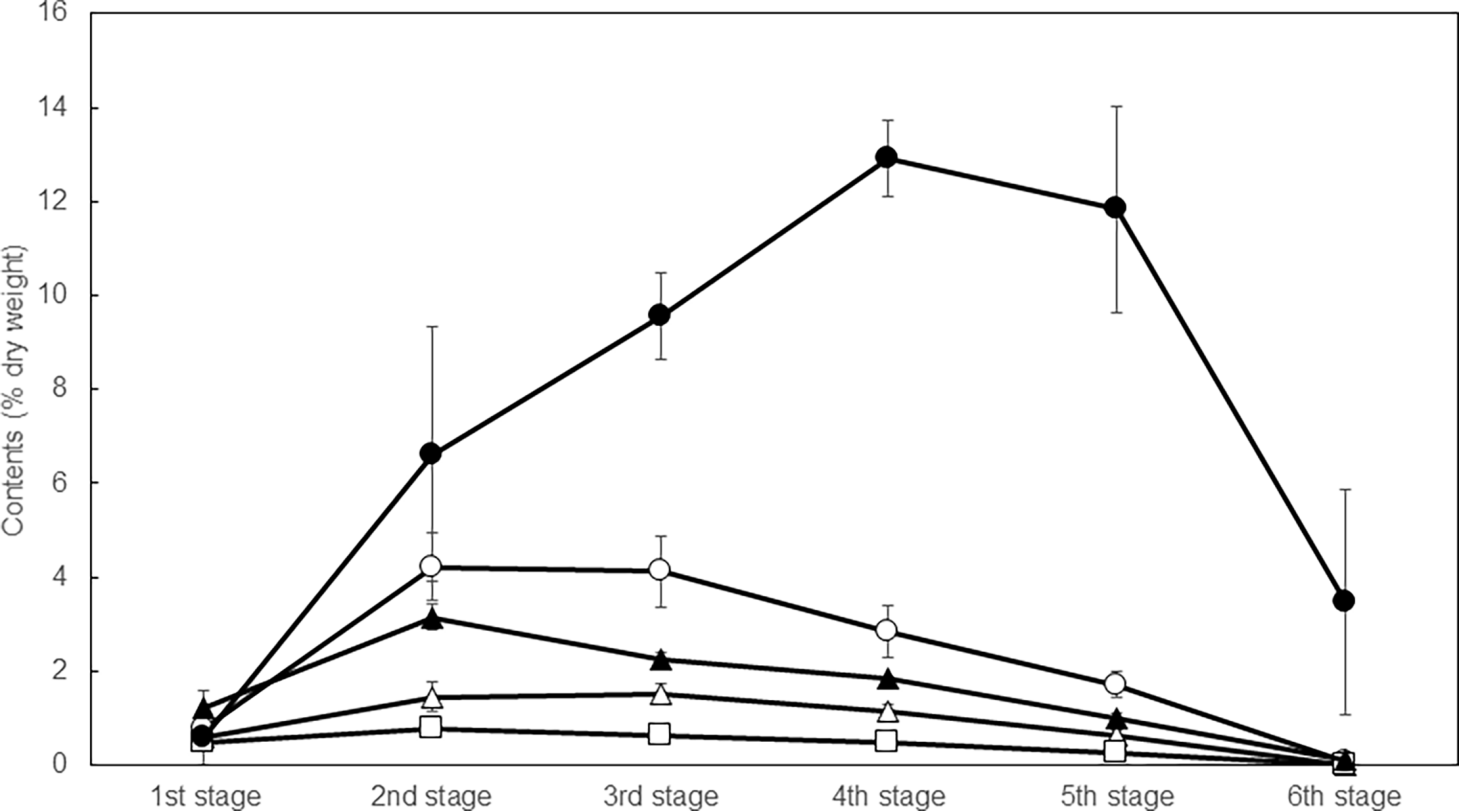
Changes in contents of major components in sesame leaves at different growth stages. Plots of the figure presents lamalbid (**I1**, open triangle), sesamoside (**I2**, open circle), shanzhiside methyl ester (**I3**, open square), pedaliin (**P4**, solid triangle) and acteoside (**P5**, solid circle).

The contents were examined at six different growth stages of the plants to determine optimum harvest stage of sesame leaves. Fig 3 shows changes in the content of major extracted components in the leaves during growth in a greenhouse. Interestingly, the acteoside (**P5**) content in the leaves increased remarkably during growing, and reached a maximum of 12.9% at the 4th stage (plant height, 74.5±9.7 cm). On the other hand, the iridoid (**I1–I3**) and pedaliin (**P4**) content in the leaves increased slightly up to the 2nd stage, and afterward decreased moderately.

Acteoside is one of the phenylethanoid glycosides, which are widely distributed in the plant kingdom; most have been isolated from medicinal plants. Studies have shown that acteoside has various biological activities, such as antioxidative, anti-inflammatory [34], hepatoprotective [35] and neuroprotective [36] activities. Recent studies have also shown that acteoside would be a potential therapeutic compound for Alzheimer’s [37,38] and Parkinson’s [39] diseases. On the other hand, it has been pointed out that many issues remain unresolved with respect to effective clinical applications of acteoside; large scale evidence-based human study and mass production for pharmaceutical applications [40].

He *et al*. [41] reported that more than 150 plant species belonging to 20 families and 77 genera contain acteoside. The acteoside content varies within plant species of the same family or genus, and high content has been reported in the leaves of *Verbascum nigrum* (3.03%) and *Verbascum xanthophoenicem* (1.58%) [42], the stems of *Cistanche tubulosa* (0.86–2.54%) and *Cistanche desericola* (0.48–2.11%) [43,44], and the aerial parts of *Plantago lanceolata* (2.17%) [45]. To the best of our knowledge, the accumulation of up to 12.9% acteoside in the leaves of *S*. *indicum* is one of the highest reported amounts in plants. It is interesting that the content in the leaves reached a maximum when the flowers began to open (4th stage), and then decreased to 3.45% at the 6th stage. This raises the question of why sesame synthesizes so much acteoside and what the accumulated acteoside is used for. The biosynthetic pathway of acteoside has still not been fully elucidated; in particular, several key enzymes and the genes encoding them are still undiscovered [40]. On the other hand, the early steps are known; acteoside is biosynthesized from tyrosine and phenylalanine through caffeic acid and hydroxytyrosol [46,47]. In this study, no caffeic acid or hydroxytyrosol was detected in any of the harvested leaves. Further qualitative and quantitative analyses of different parts of whole plants of *S*. *indicum* are therefore in progress to learn more about the biosynthesis of acteoside.

## Conclusions

Young sesame leaves contain three iridoids (**I1–I3**) and eight polyphenols (**P1–P8**). **P3** was a new compound and **I1**, **I3**, **P2** and **P8** were found in a *Sesamum* species for the first time. Acteoside (**P5**) makes a major contribution to the biological activity of young sesame leaves based on its amount and the activity of individual identified compounds. When major components were examined at six growth stages, the acteoside content in the leaves increased remarkably during growth and reached a maximum of 12.9% at the 4th stage. This is one of the highest levels reported in plants in nature. Our results indicate that the amount of compounds in the leaves of *S*. *indicum* L. depends on the growth stage, and the quantification of acteoside and the stage of the leaves at harvest should be considered when young sesame leaves are desired with high biological activity as a health food supplement, or for other end-use applications of adult or old sesame leaves as a natural source of acteoside.

## Supporting information

S1 Fig Variation in topsoil temperature throughout the experiment. The thick line indicates daily maximum temperature and thin line indicates daily minimum temperature.
(TIF)

S2 Fig Sesame plants at the studied growth stages. (TIF)

S3 Fig NMR spectra of pedalitin-6-*O*-laminaribioside (P3). (TIF)

S4 Fig Typical HPLC patterns of young sesame leaves cultivated in different regions. (TIF)

S1 Table Fresh and dry weight and number of leaves at different growth stages. (DOCX)

S2 Table NMR spectroscopic data for compounds I1, I2 and I3. (DOCX)

S3 Table NMR spectroscopic data for compounds P1 and P8. ^a^ Our previous report [12]. ^b^ The value in box brackets shows signal data in α-forms of **P1**-gluose moiety.
(DOCX)
