# Supplementary figures and images for: Chemical characterization and biological activity in young sesame leaves (Sesamum indicum L.) and changes in iridoid and polyphenol content at different growth stages
Source: PLoS One. 2018 Mar 27;13(3):e0194449. doi: 10.1371/journal.pone.0194449 (PMC5870955; doi:10.1371/journal.pone.0194449)

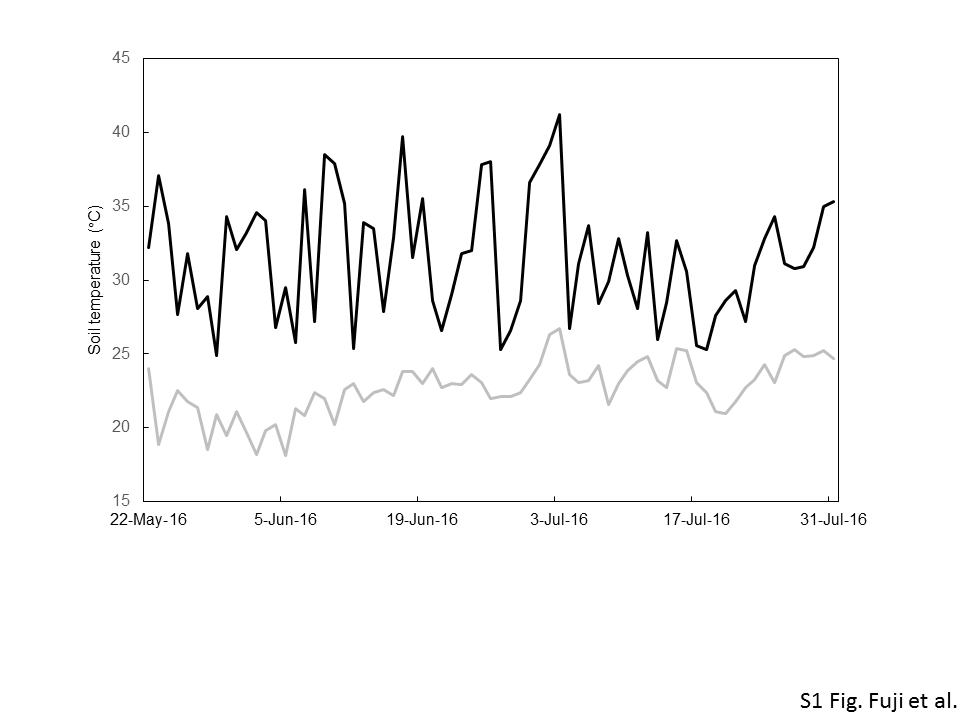

Supplement: S1 Fig — The thick line indicates daily maximum temperature and thin line indicates daily minimum temperature. (TIF) [file pone.0194449.s001.TIF]

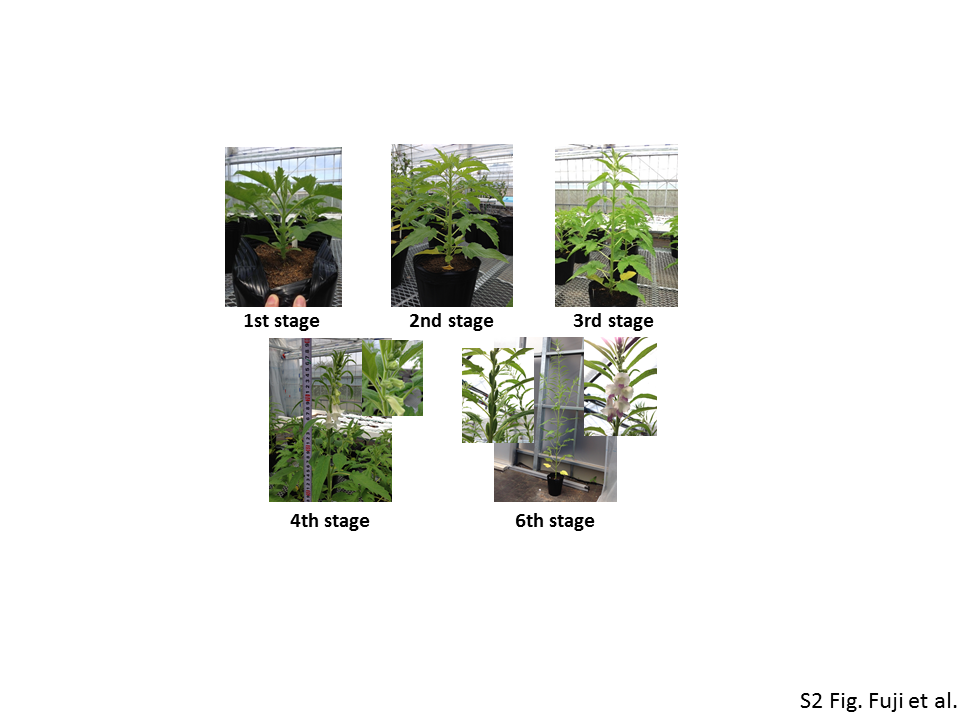

Supplement: S2 Fig — (TIF) [file pone.0194449.s002.TIF]

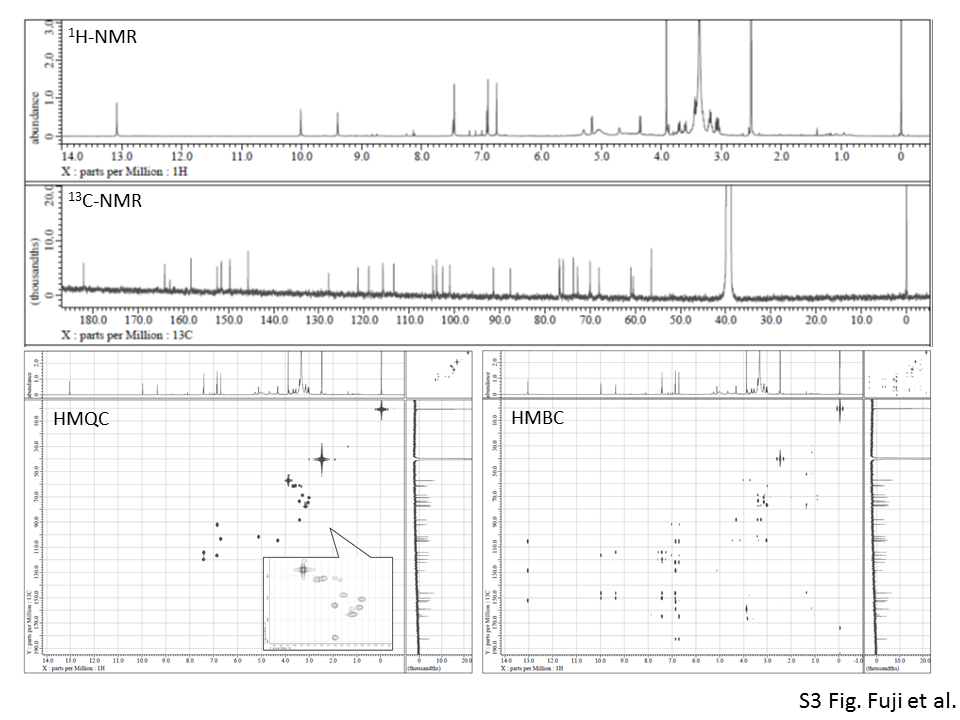

Supplement: S3 Fig — (TIF) [file pone.0194449.s003.tif]

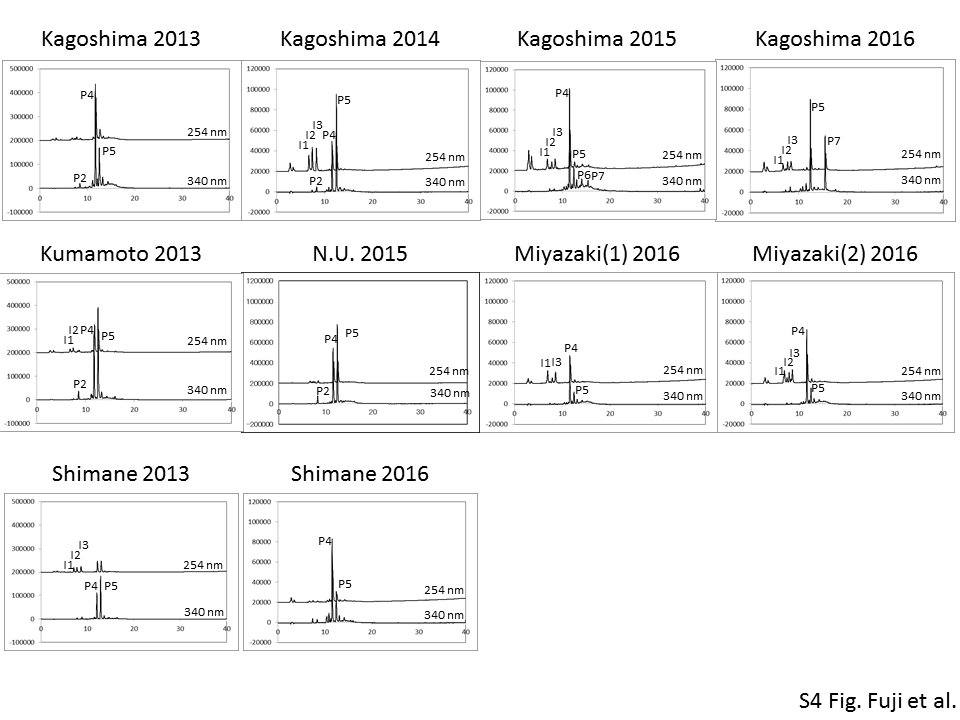

Supplement: S4 Fig — (TIF) [file pone.0194449.s004.tif]
