# Supplementary material for: Chemical characterization and biological activity in young sesame leaves (Sesamum indicum L.) and changes in iridoid and polyphenol content at different growth stages
Source: PLoS One. 2018 Mar 27;13(3):e0194449. doi: 10.1371/journal.pone.0194449 (PMC5870955; doi:10.1371/journal.pone.0194449)
Supplement: S1 Table — (DOCX) [file pone.0194449.s005.docx]

**S1 Table.** **Fresh and dry weight and number of leaves at different growth stages.**

|  | Fresh weight (g/plant) | Dry weight (g/plant) | Number of leaves (/plant) |
| --- | --- | --- | --- |
| 1st stage | 6.3 ± 1.9 | 0.95 ± 0.33 | 12.1 ± 1.5 |
| 2nd stage | 29.2 ± 2.7 | 6.35 ± 0.39 | 17.1 ± 2.8 |
| 3rd stage | 52.9 ± 5.8 | 10.7 ± 1.24 | 25.8 ± 1.1 |
| 4th stage | 49.3 ± 3.6 | 10.4 ± 0.69 | 35.3 ± 6.5 |
| 5th stage | 59.7 ± 7.6 | 13.1 ± 2.97 | 32.1 ± 5.5 |
| 6th stage | 60.8 ± 7.9 | 15.0 ± 2.14 | 105.1 ± 25.3 |
