# Supplementary material for: Chemical characterization and biological activity in young sesame leaves (Sesamum indicum L.) and changes in iridoid and polyphenol content at different growth stages
Source: PLoS One. 2018 Mar 27;13(3):e0194449. doi: 10.1371/journal.pone.0194449 (PMC5870955; doi:10.1371/journal.pone.0194449)
Supplement: S2 Table — (DOCX) [file pone.0194449.s006.docx]

**S2 Table. NMR spectroscopic data for compounds I1, I2 and I3.**

| pos. | **I1**, lamalbid | | **I2**, sesamoside | | **I3**, shanzhiside methyl ester | |
| --- | --- | --- | --- | --- | --- | --- |
|  | δ _C_ | δ _H_ (mult, *J* in Hz) | δ _C_ | δ _H_ (mult, *J* in Hz) | δ _C_ | δ _H_ (mult, *J* in Hz) |
| 1 | 94.9 | 5.62 (d,1.5) | 96.9 | 5.47 (d, 8.6) | 94.8 | 5.57 (d, 3) |
| 3 | 152.9 | 7.40 (d, 1) | 155.5 | 7.56 (s) | 152.8 | 7.40 (d, 1) |
| 4 | 111.8 | - | 113.1 | - | 111.4 | - |
| 5 | 37.7 | 2.92 (brd, 10.5) | 75.0 | - | 41.4 | 2.99 (brd, 10) |
| 6 | 78.01 | 3.95 (brt, 4) | 77.6 | 4.30 (d, 1.7) | 77.5 | 4.04 (m) |
| 7 | 77.98 | 3.54 (d, 4) | 66.0 | 3.44 (brs) | 49.2 | 1.82 (brd, 13) |
|  |  |  |  |  |  | 2.01 (brd, 13) |
| 8 | 78.7 | - | 63.8 | - | 79.0 | - |
| 9 | 49.4 | 2.80 (brd, 10.5) | 54.4 | 2.50 (d, 8.6) | 51.8 | 2.61 (dd, 3, 10) |
| 10 | 22.1 | 1.20 (s) | 17.8 | 1.48 (s) | 24.7 | 1.25 (s) |
| 11COO | 169.5 | - | 169.1 | - | 169.7 | - |
| OCH_3_ | 51.9 | 3.72 (s) | 52.3 | 3.72 (s) | 51.9 | 3.73 (s) |
| Glc-1' | 99.8 | 4.60 (d, 8) | 100.0 | 4.70 (d, 8) | 99.8 | 4.62 (d, 7.5) |
| 2' | 74.7 | 3.16 (dd, 8, 9) | 74.7 | 3.22 (dd, 8, 9) | 74.7 | 3.26 (brd, 9) |
| 3' | 78.4 | 3.34 (brd, 9) | 77.8 | 3.36 (brd, 9) | 78.0 | 3.35 (brd, 9) |
| 4' | 71.6 | 3.25 (brd, 9) | 71.8 | 3.19 (brd, 9) | 71.7 | 3.17 (brd, 9) |
| 5' | 79.0 | 3.31 (m) | 78.8 | 3.29 (m) | 78.4 | 3.31 (m) |
| 6' | 62.9 | 3.65 (dd, 6.5, 12) | 63.1 | 3.59 (dd, 7, 12) | 62.9 | 3.65 (dd, 6, 12) |
|  |  | 3.89 (dd, 2.5, 12) |  | 3.90 (dd, 1.7, 12) |  | 3.89 (2, 12) |
