# Supplementary material for: Chemical characterization and biological activity in young sesame leaves (Sesamum indicum L.) and changes in iridoid and polyphenol content at different growth stages
Source: PLoS One. 2018 Mar 27;13(3):e0194449. doi: 10.1371/journal.pone.0194449 (PMC5870955; doi:10.1371/journal.pone.0194449)
Supplement: S3 Table — a Our previous report [12]. b The value in box brackets shows signal data in α-forms of P1-gluose moiety. (DOCX) [file pone.0194449.s007.docx]

**S3 Table. NMR spectroscopic data for compounds P1 and P8.**

| pos. |  | **P1**, cistanoside F | | **P8**, martynoside | | **P5**, acteoside^a^ | |
| --- | --- | --- | --- | --- | --- | --- | --- |
|  |  | δ _C_ | δ _H_ (mult, *J* in Hz) | δ _C_ | δ _H_ (mult, *J* in Hz) | δ _C_ | δ _H_ (mult, *J* in Hz) |
| 1 |  |  |  | 132.9 |  | 131.5 |  |
| 2 |  |  |  | 117.1 | 6.74 (d, 1.5) | 117.1 | 6.69 (d, 2.0) |
| 3 |  |  |  | 147.6 |  | 146.2 |  |
| 4 |  |  |  | 147.4 |  | 144.7 |  |
| 5 |  |  |  | 112.9 | 6.82 (d, 7.7) | 116.3 | 6.67 (d, 8.5) |
| 6 |  |  |  | 121.2 | 6.69 (dd, 1.5, 7.7) | 121.2 | 6.56 (dd, 2.0, 8.5) |
| α |  |  |  | 72.13 | 3.73 (m) | 72.3 | 3.70 (m) |
|  |  |  |  |  | 4.04 (m) |  | 4.04 (m) |
| β |  |  |  | 36.6 | 2.82 (dt, 2.3, 6.9) | 36.6 | 2.79 (m) |
| OCH_3_ |  |  |  | 56.51 | 3.81 (s) | - | - |
| 1' |  | 127.7 | - | 127.7 | - | 127.7 | - |
| 2' |  | 115.2 | 7.06 (d, 2.0) | 111.8 | 7.19 (d, 1.6) | 115.2 | 7.05 (d, 2.0) |
| 3' |  | 149.8 | - | 149.4 | - | 146.9 | - |
| 4' |  | 146.9 | - | 150.8 | - | 149.9 | - |
| 5' |  | 116.5 | 6.78 (d, 8.0) | 116.5 | 6.81 (d, 7.7) | 116.5 | 6.77 (d, 8.5) |
| 6' |  | 123.2 | 6.96 (dd, 2.0, 8.0) | 124.4 | 7.08 (brd, 7.7) | 123.3 | 6.95 (dd, 2.0, 8.5) |
| α' |  | 114.9 [114.7]^b^ | 6.28 (d, 16) | 115.1 | 6.37 (d, 16) | 114.7 | 6.27 (d, 16) |
| β' |  | 147.9 [148.0] | 7.59 (d, 16) | 147.9 | 7.66 (d, 16) | 148.1 | 7.59 (d, 16) |
| C=O |  | 168.4 [168.3] | - | 168.3 | - | 168.3 | - |
| OCH_3_ |  | - | - | 56.47 | 3.88 (s) | - | - |
| Glc-1'' |  | 98.2 [94.1] | 4.55 (d, 7.7) [5.11 (d, 3.8)] | 104.2 | 4.38 (d, 8.4) | 104.2 | 4.37 (d, 8.0) |
| 2'' |  | 77.4 [74.8] | 3.32 (brt, 9.2) [3.56 (m)] | 76.2 | 3.40 (t, 8.4) | 76.1 | 3.39 (dd, 8.5, 9.0) |
| 3'' |  | 81.7 [79.2] | 3.80 (t, 9.2) [4.05 (t, 9.5)] | 81.6 | 3.83 (m) | 81.7 | 3.81 (t, 9.0) |
| 4'' |  | 70.7 [70.8] | 4.91 (m) [4.94 (m)] | 70.6 | 4.92 (t, 9.2) | 70.6 | 4.92 (t, 9.5) |
| 5'' |  | 76.2 [71.3] | 3.52 (m) [4.00 (m)] | 76.0 | 3.52 (m) | 76.3 | 3.51 (m) |
| 6'' |  | 62.5 [62.4] | 3.52 (m) [3.53 (m)] | 62.4 | 3.52 (m) | 62.4 | 3.52 (m) |
|  |  |  | 3.59 (m) [3.59 (m)] |  | 3.62 (m) |  | 3.62 (m) |
| Rhm-1''' |  | 103.1 [103.2] | 5.13 (d, 1.5) [5.19 (d, 1.5)] | 103.0 | 5.20 (brs) | 103.1 | 5.18 (d, 2.0) |
| 2''' |  | 72.4 | 3.93 (m) | 72.4 | 3.92 (m) | 72.4 | 3.91 (dd, 2.0, 3.5) |
| 3''' |  | 72.1 | 3.56 (m) | 72.07 | 3.59 (m) | 72.1 | 3.57 (dd, 3.5, 9.0) |
| 4''' |  | 73.9 [73.8] | 3.29 (m) | 73.8 | 3.30 (m) | 73.8 | 3.29 (t, 9.0) |
| 5''' |  | 70.4 [70.5] | 3.56 (m) | 70.4 | 3.56 (m) | 70.5 | 3.53 (m) |
| 6''' |  | 18.5 | 1.10 (d, 6.9) | 18.5 | 1.10 (d, 6.9) | 18.5 | 1.09 (d, 6.5) |

^a^ Our previous report [12]. ^b^ The value in box brackets shows signal data in α-forms of **P1**-gluose moiety.
